# Integrated Plasma and Glial Cell Evidence Indicates a Functional Role for hsa-miR-342-5p in Spinocerebellar Ataxia Type 7 and Its Potential Use as a Biomarker

**DOI:** 10.3390/ijms27020683

**Published:** 2026-01-09

**Authors:** Verónica M. Borgonio-Cuadra, Aranza Meza-Dorantes, José Manuel Rodríguez-Pérez, Ian A. García-Aguirre, Nadia Mireya Murillo-Melo, Nonanzit Pérez-Hernández, Oscar Hernández-Hernández, Marcela Hernández-Ortega, Zazil Herrera-Carrillo, Bulmaro Cisneros, Jonathan J. Magaña

**Affiliations:** 1 Laboratory of Genomic Medicine, Department of Genetics, National Rehabilitation Institute (INR-LGII), Ciudad de México 14389, Mexico; vborgoni@yahoo.com.mx (V.M.B.-C.); nmurillo@inr.gob.mx (N.M.M.-M.); ohernandez@inr.gob.mx (O.H.-H.); 2 Centro de Investigación en Ciencias de la Salud (CICSA), Facultad de Ciencias de la Salud, Universidad Anáhuac México, Huixquilucan 52786, Mexico; zazil.herrera@anahuac.mx; 3 Department of Bioengineering, School of Engineering and Sciences, Tecnologico de Monterrey, Campus Ciudad de México, Ciudad de México 14380, Mexico; a01730904@tec.mx (A.M.-D.); ian.garcia@tec.mx (I.A.G.-A.); 4 Departamento de Biología Molecular, Instituto Nacional de Cardiología Ignacio Chávez, Ciudad de México 14080, Mexico; josemanuel_rodriguezperez@yahoo.com.mx (J.M.R.-P.); unicanona@yahoo.com.mx (N.P.-H.); 5 Escuela Militar de Graduados de Sanidad, Secretaria de la Defensa Nacional, Miguel Hidalgo, Ciudad de México 11200, Mexico; marcelahdzor@gmail.com; 6 Department of Genetics and Molecular Biology, Center of Research and Advanced Studies (CINVESTAV-IPN), Ciudad de México 07360, Mexico

**Keywords:** circulating miRNAs, spinocerebellar ataxia type 7, plasma biomarker, human glia cells, polyQ disease

## Abstract

Spinocerebellar ataxia type 7 (SCA7) is a neurodegenerative disease characterized by cerebellar ataxia and retinal degeneration, caused by an abnormal expansion of CAG repeats at the *ATXN7* gene. Disease onset and progression vary among patients, underscoring the need for novel tools to improve disease monitoring. Circulating miRNAs represent a promising prognostic tool, due to their minimally invasive sampling and high stability. The aim of this study was to assess the expression of twelve circulating miRNAs associated with neurodegeneration in plasma samples from SCA7 patients and in an inducible SCA7 glial cell model. A comparison of SCA7 patients and controls revealed that nine miRNAs exhibited significantly higher expression. Furthermore, comparison of patients with different SCA7 phenotypes to controls revealed that most miRNAs were overexpressed in plasma from early-onset patients corresponding to the clinically more severe phenotype. Regarding the cell model, we identified three miRNAs that were dysregulated; however, only hsa-miR-342-5p displayed a pattern consistent with that observed in the plasma of patient. Our findings indicate that hsa-miR-342-5p is differentially expressed in the plasma of patients and the SCA7 cellular model, implying that it can serve as a biomarker and facilitate the identification of novel processes involved in SCA7.

## 1. Introduction

Spinocerebellar ataxia type 7 (SCA7) is an inherited neurodegenerative disorder caused by a dynamic mutation of the CAG trinucleotide located in the coding region of the *ATXN7* gene. The increased number of CAG repeats leads to the translation of a protein that contains an expanded array of glutamine residues (Poly Q). This, in turn, results in a gain of function for the mutant protein [1].

The disease has characterized primarily by progressive dysfunction of the cerebellum and brain stem, which underlies the motor and coordination deficits. A hallmark clinical characteristic that distinguishes SCA7 from other spinocerebellar ataxias is the occurrence of retinal degeneration, which results in irreversible retinopathy [1,2]. Despite the fact that SCA7 is regarded as an orphan disorder, with an estimated global prevalence of approximately 1 in 100,000 individuals, it accounts for 2–4% of all SCA cases, and its prevalence varies substantially across populations and geographic regions. Notably, higher than expected frequencies have been reported in specific populations, including those of Norway and Mexico [3].

Similar to other polyQ diseases, SCA7 exhibits genetic anticipation, whereby successive generations display earlier disease onset and increased severity due to the expansion of the CAG repeat tract across generations [4]. Several studies have indicated that the phenotypic presentation of SCA7 is associated with the number of CAG repeats and age at onset. Adult-onset SCA7 (classic and late-onset) has commonly been reported in individuals with approximately 36–47 CAG repeats [5]. In contrast, the juvenile and infantile forms (early-onset) are associated with larger expansions that often exceed 47 repeats. These forms tend to progress more rapidly, with visual impairment sometimes preceding cerebellar ataxia [1]. The childhood form is the most severe and has been linked to expansions that are very large, leading to multisystem involvement, including cardiac, renal, and muscular complications that may result in death in early life [6]. These genotype-phenotype tendencies are approximate and variable, reflecting the heterogeneity of dynamic repeat expansions.

Ataxin-7 has been described as an essential component of the STAGA complex, which mediates chromatin remodeling and transcriptional regulation through histone modification [7], underscoring its critical role in transcriptional control. The presence of mutant ataxin-7 profoundly disrupts STAGA complex activity in the human retina, leading to altered expression of the cone-rod homeobox (*CRX*) transcription factor and compromising the transcription of genes essential for photoreceptor function [8,9]. Although the full spectrum of ataxin-7 physiological functions has not yet been elucidated, evidence also supports its involvement in microtubule stabilization, cytoskeletal organization, vesicular trafficking, and the regulation of the ubiquitin–proteasome system and autophagy [10,11,12,13]. Interestingly, the gain or loss of function of the mutant ataxin-7 protein affects various cellular processes, including altered protein function due to misfolding; formation of toxic oligomeric complexes; aberrant neuronal signaling; mitochondrial dysfunction; altered axonal transport; impaired cellular protein homeostasis; RNA toxicity; and transcriptional deregulation [14]. While the molecular mechanisms underlying SCA7 remain unclear, accumulating evidence indicates that transcriptional deregulation plays a central role in SCA7 pathogenesis. Recent reports have shown that SCA7 involves the dysregulation of non-coding RNA expression, particularly miRNAs [15]. miRNAs are endogenous small RNAs that are ~20–25 nucleotides long and modulate gene expression post-transcriptionally by promoting mRNA degradation or translational repression [16]. These molecules are essential regulators of key cellular processes, including metabolism, apoptosis, stress responses, proliferation, differentiation, and nervous system development. This highlights their potential relevance in SCA7 pathogenesis [16,17,18,19].

In recent work, Borgonio-Cuadra et al. identified a circulating miRNA signature in SCA7, highlighting four miRNAs associated with prognosis, whose expression correlated with disease severity [15]. On the other hand, they also reported several dysregulated miRNAs belonging to the miR-17~92 cluster, a master regulator of neurogenesis whose altered expression has been implicated in multiple neurodegenerative disorders. Members of this family (e.g., miR-19a, miR-18a, miR-92a, and miR-20a) modulate axonal growth, neuronal differentiation, apoptosis, and autophagy, which underscores their potential pathogenic relevance [20,21]. Since circulating miRNAs reflect physiological and pathological states, these findings support the utility of these miRNAs as candidate biomarkers for SCA7.

Therefore, the objectives of our study were to quantify the expression levels of a specific group comprising twelve circulating miRNAs in patients with SCA7 and to analyze its deregulation in a specific cellular model of SCA7 (retina). Likewise, we aimed to analyze in silico pathways to predict and relate altered circulating miRNAs to deregulated molecular pathways with SCA7. Moreover, integrating miRNA analysis with established SCA7 cellular models, including retinal-derived systems that recapitulate central nervous system (CNS) involvement, may facilitate the determination of mechanistic insights and the identification of novel therapeutic targets.

## 2. Results

### 2.1. Differential Expression of Circulating miRNAs in Patients with SCA7

For this new cohort analysis, a total of 32 participants were included (16 patients with SCA7 and 16 healthy controls). The study groups were matched based on age and sex. As anticipated, the patient cohort with SCA7 exhibited clinical characteristics consistent with this polyQ disease, a finding corroborated by the presence of more than 36 repeated CAGs identified in the molecular diagnosis [22]. Table 1 illustrates the primary demographic characteristics of the population under study.

The study cohort comprised a total of 16 patients, including 8 individuals with an adult-onset (AO) phenotype and 8 individuals with an early-onset (EO) phenotype.

**Figure 1:**
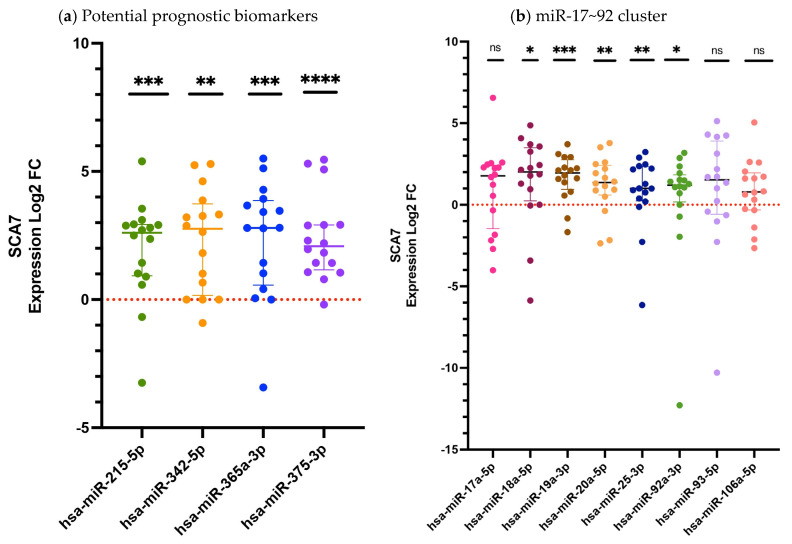
The relative expression of circulating miRNAs in the plasma of patients with SCA7 (Log2 FC). (**a**) Expression levels of potential prognostic candidate miRNAs; (**b**) The expression levels of miRNAs belonging to the miR-17~92 cluster. The quantification was performed by RT-qPCR with hsa-miR-483 serving as an endogenous control. The data set, encompassing twelve miRNAs, is presented as medians and interquartile ranges. Results are shown as log2 fold change (Log2FC), where positive and negative values indicate increase or decrease relative expression to the reference group. Log2FC greater than 1 is considered as change. The expression differences between the SCA7 group and the control group were determined by the Mann–Whitney U test. A *p*-value < 0.05 was considered significant, asterisks denote significant differences between patients with SCA7 and controls (* *p* < 0.05; ** *p* < 0.01; *** *p* < 0.001; **** *p* < 0.0001). The study included 16 subjects in each group. ns = no significant.

The plasma samples from SCA7 and from a matched control group were independently evaluated by quantitative real-time polymerase chain reaction (qRT-PCR) to assess the differential relative expression (log2FC) of the twelve candidate circulating miRNAs (Figure 1). Of these, four have been previously identified as putative prognostic biomarker candidates (Figure 1a), while the remaining eight belong to the miR-17~92 cluster (Figure 1b), have been described as important players in neurodegeneration (see Section 4 for miRNAs selection criteria used in this study).

A subsequent comparison of patients with SCA7 and control subjects revealed that nine of the analyzed miRNAs exhibited statistically significant differences in expression. Four of these correspond to the previously proposed prognostic biomarker candidates (hsa-miR-215-5p, *p* = 0.0001; hsa-miR-342-5p, *p* = 0.0038; hsa-miR-365a-3p, *p* = 0.0001; and hsa-miR-375-3p, *p* < 0.0001) (Figure 1a), while five miRNAs from the miR-17~92 cluster (hsa-miR-18a-5p, *p* = 0.0135; hsa-miR-19a-3p, *p* = 0.0001; hsa-miR-20a-5p, *p* = 0.0019; hsa-miR-25-3p, *p* = 0.0019; and hsa-miR-92a-3p, *p* = 0.0124) also showed significant deregulation (Figure 1b), consistent with previous reports [15]. Conversely, the remaining three miRNAs (hsa-miR-17a-5p, hsa-miR-93-5p, and hsa-miR-106a-5p) demonstrated no statistically significant alterations (Figure 1b).

**Figure 2:**
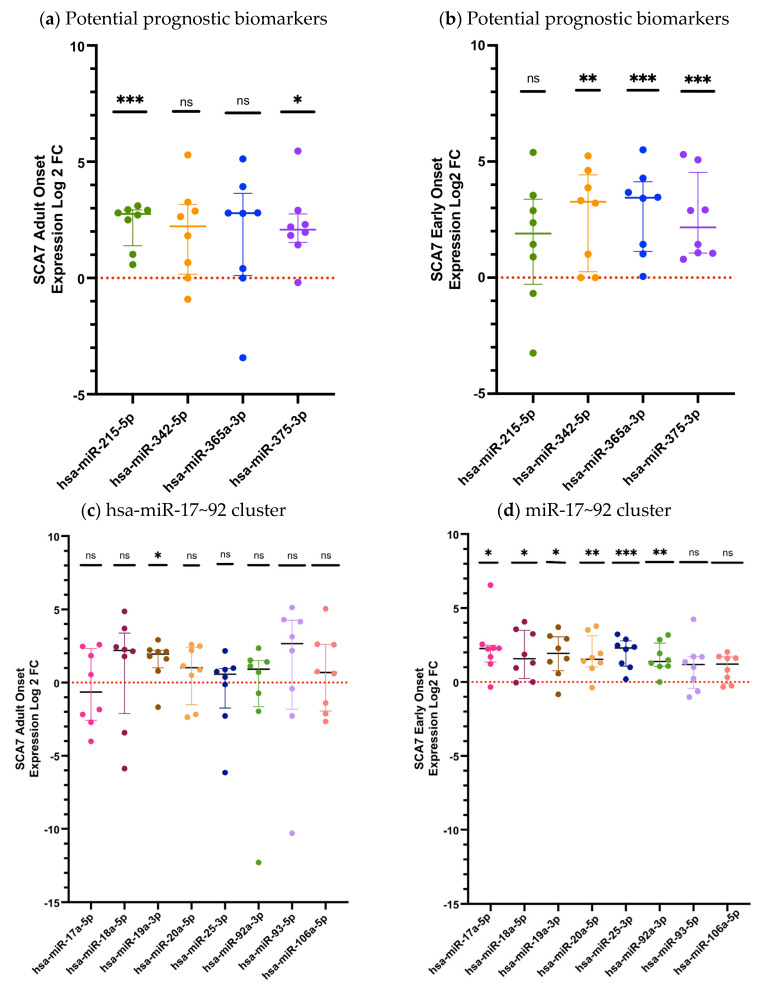
The relative miRNA expression in the plasma of patients with the Adult Onset (AO) and Early Onset (EO) SCA7 phenotypes. (**a**) Expression levels of potential prognostic candidate miRNAs in AO patients; (**b**) Expression levels of prognostic candidate miRNAs in EO patients; (**c**) Expression levels of miRNAs belonging to the miR-17~92 cluster in AO patients. (**d**) Expression levels of miRNAs belonging to the miR-17~92 cluster in EO patients. The quantification was performed using RT-qPCR, with hsa-miR-483 serving as an endogenous control. The data for the three miRNAs analyzed is shown as medians and interquartile ranges. Results are shown as log2 fold change (Log2FC), where positive and negative values indicate an increase or decrease in relative expression to the reference group. Log2FC greater than 1 is considered as change. The expression differences between the SCA7 group and the control group were determined by the Mann–Whitney U test. A *p*-value of *p* < 0.05 was considered significant; asterisks denote significant differences between patients with SCA7 and controls (* *p* < 0.05; ** *p* < 0.01; *** *p* < 0.001). ns = no significant.

Subsequently, we analyzed the relative expression of the twelve deregulated circulating miRNAs in the subjects with the pathological phenotypes Early Onset (EO) and Adult Onset (AO) (Figure 2). When we analyzed expression levels in patients with the EO phenotype, we observed that nine miRNAs (hsa-miR-342-5p, *p* = 0.0083; hsa-miR-365a-3p, *p* = 0.0003; hsa-miR-375-3p, *p* = 0.0002; hsa-miR-17-5p, *p* = 0.0104; hsa-miR-18a-5p, *p* = 0.0145; hsa-miR-19a-3p, *p* = 0.0107; hsa-miR-20a-5p, *p* = 0.0099; hsa-miR-25-3p, *p* = 0.0002; and hsa-miR-92a-3p, *p* = 0.0048) were significantly dysregulated, whereas three (hsa-miR-215-5p, *p*= 0.1488; hsa-miR-93-5p, *p* = 0.1304; and hsa-miR-106a-5p *p* = 0.1304) showed no significant differences, compared with controls (Figure 2a,c). In contrast, the AO group displayed differential expressions in only three miRNAs (hsa-miR-215-5p, *p* = 0.0002; hsa-miR-375-3p, *p* = 0.0104; and hsa-miR-19a-3p, *p* = 0.0104) while the remaining miRNAs exhibited no detectable dysregulation *p* > 0.05 (Figure 2b,d). These findings suggest a more pronounced and broader miRNA deregulation in EO patients relative to those with AO disease (Figure 2).

**Figure 3:**
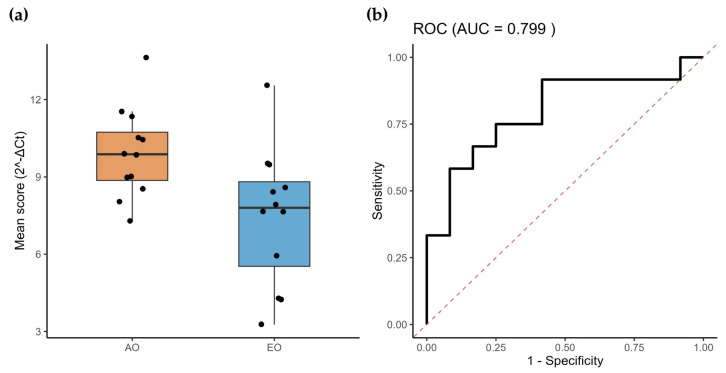
miRNAs associated with SCA7 severity. (**a**) Mean expression of the twelve miRNAs associated with SCA7 severity (AO and EO). (**b**) ROC analysis shows that the average expression of the prognostic miRNA-model accurately discriminates SCA patients from healthy controls.

Thereafter, we compared the differentially expressed miRNAs between adult-onset (AO; *n* = 8) and early-onset (EO; *n* = 8) patients. As shown in Supplementary Figure S1, nearly all analyzed miRNAs exhibited an overexpression trend in the EO group, consistent with our previous observations; however, statistically significant differences were detected only for hsa-miR-17-5p (*p* = 0.0332) and hsa-miR-25-3p (*p* = 0.0070). It is important to note that these comparison groups were heterogeneous and not matched by age or sex, which may have introduced bias into this analysis. To further clarify this effect, and given the increased clinical severity associated with the EO phenotype, we next assessed whether a subset of miRNAs could serve as prognostic biomarkers. Receiver operating characteristic (ROC) curve analysis demonstrated that the prognosis-related miRNA signature exhibited strong discriminatory performance, with an Area Under the Curve (AUC) of 0.799 (Figure 3).

We next examined the relationship between the expression levels of the identified miRNAs and key clinical and molecular variables, including CAG repeat length, age at onset, and the S factor, which integrates modifiers of disease severity in polyglutamine disorders by relating CAG repeat size to age at onset and disease duration [23]. Correlation analyses revealed that a subset of miRNAs showed significant associations with CAG repeat length and age at onset, in agreement with the composite gene module defined by the mean expression of the dysregulated miRNAs (Supplementary Table S1). Notably, when considering the entire SCA7 cohort in comparison with controls, hsa-miR-25-3p displayed consistent and significant correlations with both CAG repeat length (r = 0.6293, *p* = 0.0128) and age at onset (r = −0.6293, *p* = 0.0089). In contrast, stratified analysis of the most severe early-onset (EO) phenotype revealed stronger and more extensive associations. In this subgroup, hsa-miR-93-5p exhibited robust correlations with CAG repeat length (r = −0.8143, *p* = 0.0138) and age at onset (r = 0.8503, *p* = 0.0074). Additionally, hsa-miR-92a-3p (r = −0.7904, *p* = 0.0195) and hsa-miR-106a-5p (r = −0.7305, *p* = 0.0395) were significantly correlated with CAG repeat length. In contrast, the remaining miRNAs did not show clear or statistically significant associations with these clinical or molecular parameters. Collectively, these results indicate that specific miRNAs display stronger correlations with disease-defining variables in the EO phenotype, supporting the notion that this subset of miRNAs may reflect modifiers of disease severity. Taken together, these findings reinforce the potential utility of the identified miRNA prognostic signature as a predictor of SCA7 disease progression.

### 2.2. Validation of Plasmatic miRNAs in a Human Cellular Model of SCA7

**Figure 4:**
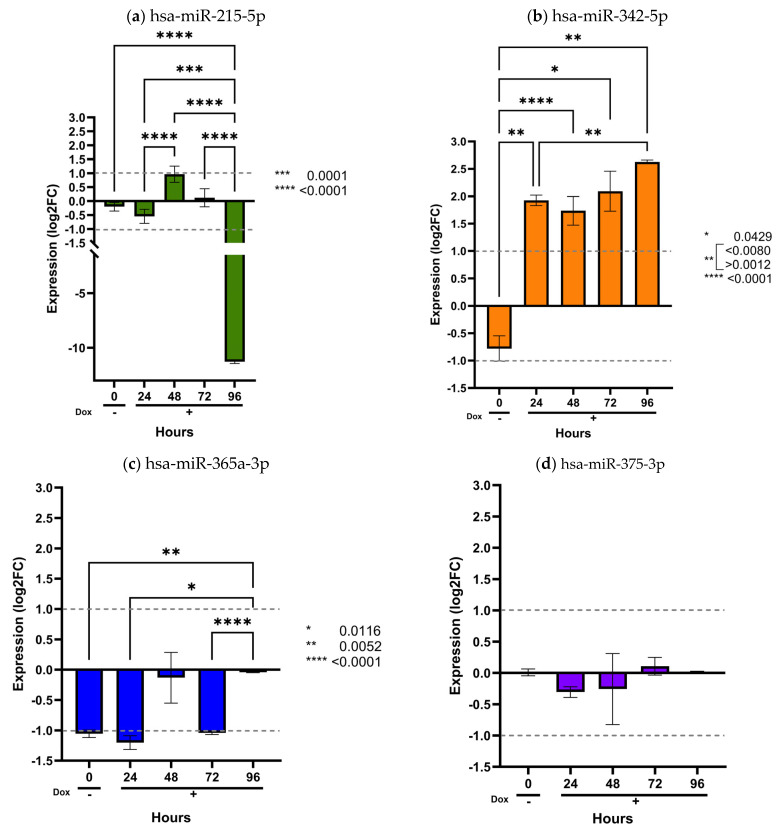
Validation of prognostic miRNAs in the inducible MIO-M1 SCA7 glial model. MIO-M1 Q10+*ATXN7* cells were used as the reference group, and MIO-M1 Q64+*ATXN7* cells were induced with (+Dox) or without (−Dox) doxycycline (1 mg/mL) for 24, 48, 72, and 96 h. Expression of the four prognostic miRNAs was quantified using the ∆∆CT method and normalized to U6, RNU44, and RNU48. Panels (**a**–**d**) display the expression profiles of hsa-miR-215-5p, hsa-miR-342-5p, hsa-miR-365a-3p, and hsa-miR-375-3p, respectively. Results are shown as log2 fold change (Log2FC), where positive and negative values indicate an increase or decrease in relative expression to the reference group. Log2FC greater than 1 is considered as change. Bars represent mean ± SD of duplicate assays. Statistical differences across time points were assessed using a repeated-measures ANOVA with Tukey post hoc comparison (*p* < 0.05). Asterisks denote significant differences between patients with SCA7 and controls (* *p* < 0.05; ** *p* < 0.01; *** *p* < 0.001; **** *p* < 0.0001).

**Figure 5:**
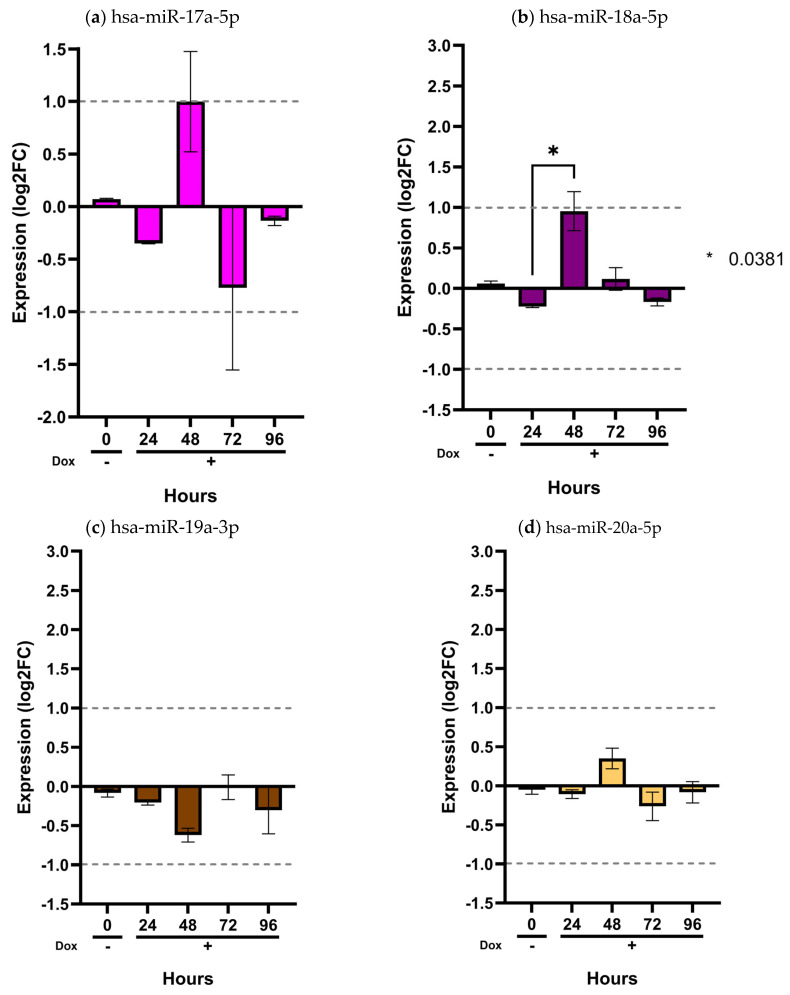

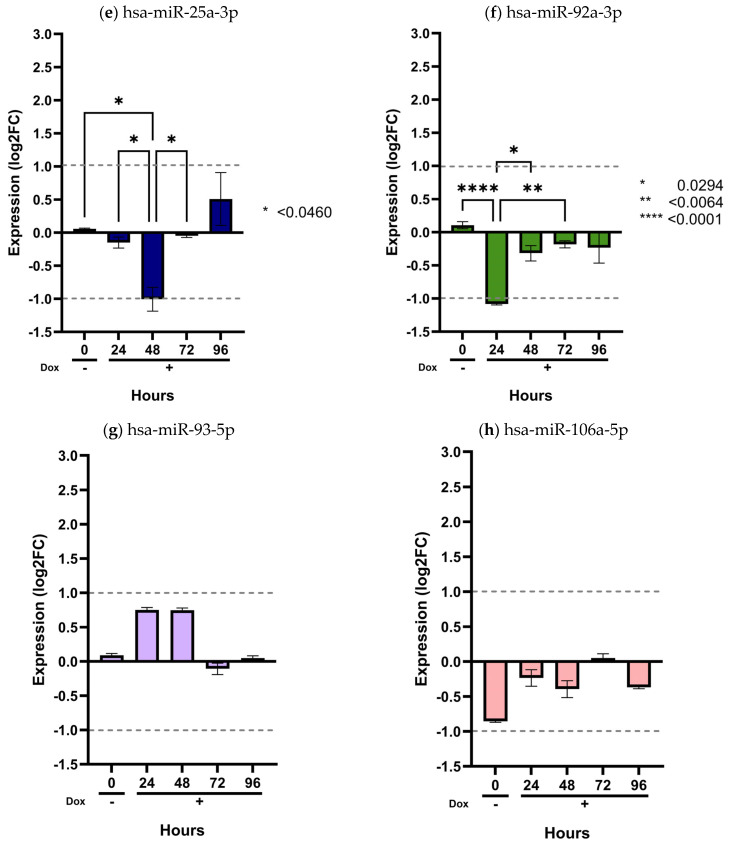
Validation of the miR-17/92 cluster in the inducible MIO-M1 SCA7 glial model. MIO-M1 Q10+*ATXN7* cells served as the reference group, while MIO-M1 Q64+*ATXN7* cells were induced with (+Dox) or without (−Dox) doxycycline (1 mg/mL) for 24, 48, 72, and 96 h. Expression of the eight miRNAs from the miR-17/92 cluster was quantified using the ∆∆CT method, normalized to U6, RNU44, and RNU48. Panels (**a**–**h**) show the expression profiles of hsa-miR-17a-5p, hsa-miR-18a-5p, hsa-miR-19a-3p, hsa-miR-20a-5p, hsa-miR-25-3p, hsa-miR-92a-3p, hsa-miR-93-5p, and hsa-miR-106a-5p, respectively. Results are shown as log2 fold change (Log2FC), where positive and negative values indicate an increase or decrease in relative expression to the reference group. Log2FC greater than 1 is considered as change. Bars represent mean ± SD of duplicate assays. Statistical differences across time points were assessed using a repeated-measures ANOVA with Tukey post hoc comparison (*p* < 0.05). Asterisks denote significant differences between patients with SCA7 and controls (* *p* < 0.05; ** *p* < 0.01; **** *p* < 0.0001).

To assess the transcriptional dysregulation of the analyzed miRNAs within the central nervous system, we evaluated their expression in an inducible human Müller glial SCA7 cell model, thereby approximating their regulatory behavior in this tissue. We previously generated a MIO-M1-derived cell line expressing human ataxin-7 with a pathological polyglutamine expansion of 64 glutamine residues (Q64) under the control of a Tet-On inducible system (MIO-M1–Q64 cells) [24,25]. The expression levels of the twelve selected miRNAs, normalized to snoRNA U6, RNU44, and RNU48, were quantified in uninduced MIO-M1–Q64 cells and after doxycycline induction at 24, 48, 72, and 96 h (Figure 4 and Figure 5), using MIO-M1–Q10 cells with normal polyglutamine expansion of 10 glutamine residues (Q10) as the control.

As shown in these figures, only two miRNAs exhibited detectable dysregulation over time. Initially, hsa-miR-215-5p displayed a slight increase in expression at 48 h, followed by a marked decrease at 96 h (*p* < 0.0001) (Figure 4a). However, this temporal pattern did not correlate with the expression levels observed in plasma samples from SCA7 patients.

In contrast, hsa-miR-342-5p showed a gradual and sustained increase beginning at 24 h and continuing through 96 h (*p* < 0.05) (Figure 4b). Notably, this miRNA was consistently elevated in patient plasma (Figure 1a), with a significant overexpression in the early-onset phenotype (Figure 2b). The overexpression pattern of hsa-miR-342-5p in the inducible glial model mirrors that observed in patient samples, supporting the pathophysiological relevance of our in vitro findings. Conversely, the remaining ten miRNAs (hsa-miR-365a-3p, hsa-miR-375-3p, hsa-miR-17a-5p, hsa-miR-18a-5p, hsa-miR-19a-3p, hsa-miR-20a-5p, hsa-miR-2-3p, hsa-miR-92a-3p, and hsa-miR-106a-5p) showed no significant changes in expression across time points (*p* > 0.05) (Figure 4 and Figure 5).

### 2.3. Interaction of miRNA Target Genes and Enrichment Analysis

**Figure 6:**
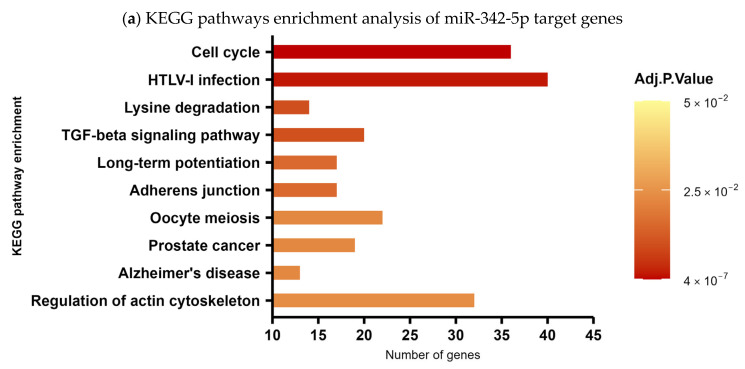

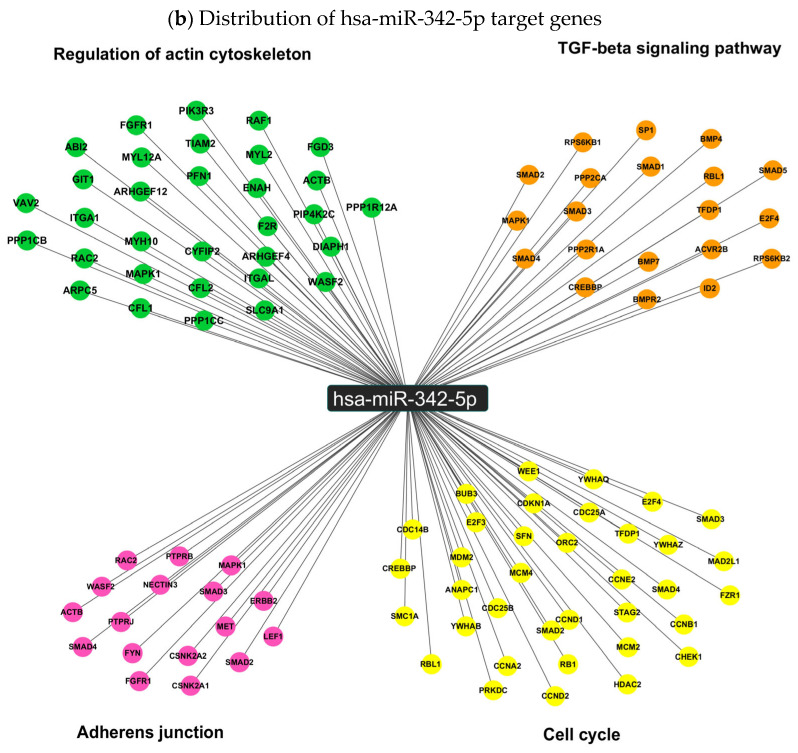
KEGG pathway enrichment analysis of hsa-miR-342-5p target genes. (**a**) Top ten enriched pathways (*p* < 0.05) associated with predicted targets of hsa-miR-342-5p. The *x*-axis indicates the number of target genes per pathway, and the *y*-axis lists the corresponding KEGG pathways. (**b**) Distribution of hsa-miR-342-5p target genes across four highlighted pathways: cell cycle, TGF-β signaling, adherens junctions, and actin cytoskeleton regulation.

In order to predict the biological processes potentially regulated by the overexpression of hsa-miR-342-5p, we performed a KEGG pathway enrichment analysis to contextualize the function of this miRNA within established biological networks and to infer its possible clinical relevance in SCA7. Target genes were identified using prediction tools based on databases of experimentally validated interactions. KEGG pathway enrichment analysis revealed that hsa-miR-342-5p is predicted to regulate a total of 1708 target genes. Figure 6a shows the top-ten enriched pathways (*p* < 0.05). Among the most prominent pathways are the cell cycle pathway with 36 targets as potential candidates (*p* ≤ 0.001), the TGF-β pathway with 20 targets (*p* = 0.01), the adherens junction pathway with 17 targets (*p* = 0.01), and the regulation of actin cytoskeleton pathway with 32 targets (*p* = 0.02) (Figure 6b).

Interestingly, within the most enriched pathways, hsa-miR-342-5p exhibited the potential to regulate a set of biologically relevant target genes involved in proliferation, differentiation, migration, and tissue maintenance. Notably, among its predicted negatively regulated targets in the cell-cycle pathway, we identified key cyclins such as CCNA2, CCNB1, and CCND1, as well as critical regulators of cell division, including CDC14B and CDC25A/B. In addition, hsa-miR-342-5p may influence cell-cycle suppression through targets such as CDKN1A (p21) and RB1 (Figure 6b). Within the TGF-β signaling pathway, hsa-miR-342-5p is predicted to modulate major components of both the canonical (Smad2, Smad3, Smad4) and non-canonical (MAPK/ERK) branches, including bone morphogenetic proteins BMP4 and BMP7, as well as their receptor BMPR2 (Figure 6b). Furthermore, in the adherens junction and actin cytoskeleton regulation pathways, hsa-miR-342-5p may target essential genes involved in cell adhesion and structural integrity, such as ACTB (β-actin), NECTIN3, CFL1 (cofilin), Raf1 and actin-modulating formins including DIAPH1 (Figure 6b).

## 3. Discussion

Clinical staging of SCA7 is currently based on subjective cerebellar and extracerebellar rating scales (SARA, BARS, INAS) [26], which are time-consuming and require extensive training to reduce bias. This underscores the need for objective, disease-specific biomarkers, ideally detectable at early or presymptomatic stages, to improve disease monitoring and clinical trial outcomes. Accordingly, several objective measures have been explored, including MRI volumetry, oculomotor parameters, gait analysis, and retinal thickness [27,28], although these approaches may be costly or limited in accessibility. Minimally invasive biomarkers derived from body fluids have therefore gained attention, encompassing genomic, transcriptomic, epigenetic, proteomic, and metabolomic markers. In SCA7, however, only a limited number of studies have addressed these strategies, with encouraging results reported for blood neurofilament light chain levels [28,29], oxidative stress markers [30], and some selected metabolites [31]. Notably, circulating miRNAs represent a particularly attractive biomarker class, as brain-derived miRNAs can cross the blood–brain barrier via exosomes or with argonaute2 interactions and are protected from degradation in biological fluids [32,33,34,35]. A variety of studies have demonstrated the presence of miRNAs within the nervous system, where they play a critical role in maintaining homeostasis and ensuring proper function [36,37]. Conversely, aberrant expressions of different miRNAs in nervous tissue have been associated with neurodegenerative diseases [38]. Furthermore, recent studies indicate that the expression of miRNAs may serve as biomarkers for diagnosis and monitoring of the progression of polyglutamine diseases, including Huntington’s disease [39] and ataxia type 3 (SCA3) [40]. In the context of SCA7, however, the role of miRNAs remains to be extensively elucidated [19,41]. In a recent report, our research group documented a set of at least 71 circulating miRNAs that exhibited altered levels in patients diagnosed with SCA7 [15]. From these, a subset of interest was selected, comprising four miRNAs previously proposed as prognostic biomarker candidates, as well as eight members of the miR-17~92 cluster. These exhibited an overexpressed pattern and have been implicated in neurodegenerative processes [20,21].

In this study, we identified nine dysregulated plasma miRNAs in patients with SCA7 compared with healthy controls, supporting the potential utility of circulating miRNAs as disease-associated biomarkers. Notably, a greater number of altered miRNAs was observed in individuals with the more severe early-onset (EO) phenotype compared with those with adult-onset (AO) disease, suggesting that the extent of miRNA dysregulation may be linked to clinical severity. A limitation of the present study is the imbalance in sex distribution, with approximately 75% of the analyzed individuals being male. This skew primarily reflects the requirement to independently match patients and controls by age and sex, together with the overrepresentation of younger affected males in our cohort, likely attributable to paternal inheritance, as patrilineal transmission has been associated with increased CAG repeat instability [42]. Although sex-related differences in SCA7 have not been clearly established, it will be important to determine whether the observed trends are consistent across sexes. In addition, stratification by clinical phenotype, while biologically informative, inevitably reduces statistical power despite the presence of clear expression trends. Accordingly, expansion of the cohort will be essential to confirm these findings and to strengthen the robustness of phenotype- and sex-related analyses. Importantly, several plasma miRNAs were exclusively deregulated in EO patients, indicating an association with distinct disease presentations and suggesting their potential utility in discriminating between EO and AO phenotypes. To further explore this observation, we performed a computational analysis of miRNAs differentially expressed between AO and EO patients, which led to the identification of a set of twelve miRNAs with putative prognostic value. This expression pattern may reflect a transition from localized neurodegeneration to broader systemic involvement as disease severity increases. Future longitudinal studies will be required to validate this hypothesis. Collectively, these findings indicate that this miRNA panel holds promise as a sensitive tool for monitoring disease progression in SCA7.

With regard to the analysis of the panel of miRNAs in the human glial model, only a subset of these miRNAs exhibited detectable dysregulation, namely, hsa-miR-215-5p and hsa-miR-342-5p, the latter demonstrating the strongest concordance with the plasma findings. The limited overlap between the in vitro and in vivo profiles likely reflects fundamental biological differences. Circulating miRNAs originate from multiple tissues and integrate systemic physiological and pathological signals [43,44], whereas the cellular model captures molecular alterations restricted to a single CNS-derived cell type [45,46]. Furthermore, the absence of intercellular communication, immune activation, and metabolic inputs in vitro may hinder the detection of miRNAs that are dysregulated exclusively within the complex in vivo environment [41,47]. Collectively, these elements provide a rationale for why only a limited proportion of circulating miRNAs maintain deregulated expression in Müller glial cells. However, the consistent and significant dysregulation of hsa-miR-342-5p in both plasma and the glial model highlights its potential as a non-invasive circulating biomarker and suggests its involvement in disease-related pathogenic pathways. Further studies using larger and geographically diverse patient cohorts, as well as functional assays, are required to clarify the role of hsa-miR-342-5p in SCA7 pathogenesis.

The current state of research on hsa-miR-342-5p in neurodegeneration is limited; however, it has been identified as being dysregulated in and closely related to neurodegeneration, particularly in Alzheimer’s disease (AD), where it acts as a key molecular regulator in the pathological processes of the disease, and is being used as a biomarker [48].

In line with the relevance of hsa-miRNA-342-5p expression in both plasma and the glial cell model, we have conducted a pathway enrichment analysis using KEGG. Four fundamental pathways were revealed: cell cycle, TGF-β signaling, adherens junctions, and cytoskeleton regulation. It is worth noting their interdependence in the context of neurodegeneration. For instance, TGF-β signaling maintains mature glial cells in a quiescent state, and its dysregulation can trigger aberrant cell-cycle re-entry. This is a well-established early mechanism of neuronal death [49,50]. Concurrently, TGF-β signaling plays a pivotal role in regulating the organization and stability of the actin cytoskeleton and the integrity of adherens junctions. Additionally, TGF-β signaling exhibits a dual function in neural physiology, supporting development and homeostasis. However, when dysregulated, it can promote inflammation, glial reactivity, and neurodegeneration [51,52,53]. Our in-depth analysis has identified several genes within these pathways, including SMAD2/3/4/5, BMP4, BMP7 [54], and a cell-cycle regulator, CCND1, as potential targets of hsa-miR-342-5p. According to our *in silico* predictions, these genes are negatively regulated by hsa-miR-342-5p. Notably, *BMP7* has been widely reported as a target of miR-342-5p during osteoblast differentiation and migration [55]. In parallel, *CCND1* has been shown to be negatively regulated by miR-342-5p in leukemia cells and in vivo models, highlighting the role of this miRNA in controlling pathways related to cellular differentiation and proliferation [56]. Disruption of adherens junctions and cytoskeletal architecture also has important pathological implications in SCA7. Junctional integrity is critical for preserving the function of the blood-brain and blood-retinal barriers. Its impairment contributes to tissue disorganization and Müller glia dysfunction [57]. In a comparable manner, abnormalities in the actin cytoskeleton have been demonstrated to impair axonal transport, synaptic stability, and cellular morphology. Importantly, CFL1 and Raf1 are key components of cell adhesion and cytoskeletal organization and have been widely described as a direct regulatory target of miR-342-5p, underscoring the involvement of this miRNA in the modulation of structural integrity and cellular dynamics [58,59]. These phenomena are commonly observed in neurodegenerative diseases [60,61,62]. Recent findings indicate a mechanistic relationship between cytoskeletal alterations and miRNA dysregulation, with oxidative stress and dysfunction of protein degradation systems, including the ubiquitin proteasome pathway, playing a role in the accumulation of misfolded proteins in neurodegenerative diseases such as Alzheimer’s disease (AD), Parkinson’s disease (PD), Huntington’s disease, and SCA7 [63,64,65]. Consequently, the dysregulation of miRNAs impacting these interconnected pathways may potentially disrupt glial homeostasis and exacerbate neurodegenerative progression in SCA7. Given their functional interdependence, alterations in any of these pathways may propagate across others, collectively contributing to glial and neuronal dysfunction in SCA7. It is worth noting that the deregulation of hsa-miR-342-5p provides a potentially fruitful avenue for elucidating the molecular mechanisms underlying SCA7.

Despite the limited size and heterogeneity of the sample, which is consistent with the rarity of SCA7 (<1/100,000), this study serves as a cohort-validation effort, incorporating newly enrolled patients to reinforce the reproducibility of results. While the cross-sectional design restricts causal interpretation, the consistency across independent samples substantiates the robustness of the observed alterations in miRNAs.

In summary, this study provides the first characterization of diverse circulating miRNAs in a clinically well-defined cohort of patients with SCA7 that corroborates their dysregulation in a human central nervous system-derived cell model, supporting a potential correlation between circulating and tissue-specific miRNA expression. Together, these findings highlight the biological relevance of these miRNAs and suggest their involvement in key molecular pathways associated with SCA7 pathogenesis. Collectively, these findings provide novel biological insights and establish a foundation for future longitudinal and mechanistic studies.

## 4. Materials and Methods

### 4.1. Study Population

An analytical, cross-sectional and observational study was designed. The study participants were recruited from the central region of the state of Veracruz in Mexico, who attended the Center of Rehabilitation and Social Inclusion of Veracruz (CRIS-VER) and were referred to the Instituto Nacional de Rehabilitación Luis Guillermo Ibarra Ibarra (INR-LGII). The present study was performed following the guidelines of the Helsinki Declaration and was approved by the Research and Ethics Committee of INR-LGII (Protocol code: 15/12). Every participant signed an informed consent voluntarily. Initially, all study participants were classified as SCA7 patients or healthy individuals through molecular diagnosis using capillary electrophoresis, as previously reported by our group [26].

The study included 16 individuals diagnosed as SCA7 who presented two clinical phenotypes and were divided into two groups: The early onset (EO) group included 8 patients with an early age of onset (<20 years old), who presented cerebellar symptoms (motor alterations), decreased visual acuity or macular dysfunction and more than CAG repeats at the *ATXN7* gene. While the adult onset (AO) group involved 8 patients with onset of symptoms in adulthood, cerebellar symptoms and the presence of 37 to 46 CAG repeats [66].

A complete questionnaire and clinical history were applied to all patients. Clinically, the patients were evaluated with neurological examinations according to the American guidelines of the Mayo Clinic [67]. The evaluation of cerebellar symptoms was determined by the Scale for the Assessment and Rating of Ataxia (SARA) [67], while the exploration of extracerebellar characteristics was performed using the Inventory of Non-Ataxia Symptoms (INAS) [68]. Every patient suspended the administration of medications and dietary supplements three months prior to the study.

SCA7 patients with ataxia secondary to alcoholism, neoplasms, vascular pathology, congenital malformations, chronic-degenerative diseases, autoimmune and inflammatory diseases, as well as other genetic alterations, were excluded. Other exclusions were: patients with infections, liver disorders, oncological conditions and pregnant women. Patients who were using drugs, pharmacological treatments, or immunosuppressants were also excluded.

The comparison group was formed by 16 healthy individuals, with a negative molecular diagnosis for SCA7; they were recruited from the same geographic region, had similar living conditions and a similar diet to the patient group. Additionally, the participants of both study groups were matched by age and sex. The exclusion criteria for the comparison group were the same as those set for patients with SCA7.

### 4.2. Plasma Collection and Separation

From every participant, 5 mL of peripheral blood was collected in Vacutainer tubes containing EDTA as an anticoagulant; the samples were centrifuged at 1900× *g*/10 min at 4 °C, the upper phase, represented by plasma, was transferred to an Eppendorf tube, and then it was centrifuged at 16,000× *g*/4 °C to remove cell debris. Finally, the plasma was recovered in an Eppendorf tube and stored at −80 °C for later use.

### 4.3. miRNA Selection

In order to explore miRNAs as markers in SCA7 we selected four possible prognosis miRNAs to SCA7 (hsa-miR-215-5p [ID000518], hsa-miR-342-5p [ID002147], hsa-miR-365a-3p [ID001020], hsa-miR-375-3p [ID000564]) [8] and eight were grouped in the 17 ~ 92 family (hsa-miR-17a-5p [ID002308], hsa-miR-18a-5p [ID002422], hsa-miR-19a-3p [ID000395], hsa-miR-20a-5p [ID000580], hsa-miR-25-3p [ID000403], hsa-miR-92a-3p [ID000431], miR-93-5p [ID000432], hsa-miR-106a-5p [ID002169]). At the in silico level, four prognosis-associated miRNAs were identified as potential prognostic biomarkers for SCA7 by comparing differentially expressed miRNAs between the early-onset (EO) and adult-onset (AO) phenotypes. These miRNAs (hsa-miR-215-5p, hsa-miR-342-5p, hsa-miR-365a-3p, and hsa-miR-375-3p) were consistently overexpressed in the more severe EO phenotype [15]. Notably, the combined expression of these miRNAs demonstrated a robust prognostic performance in a preliminary analysis, yielding an area under the ROC curve (AUC) of 0.84, which supports the potential clinical relevance of this miRNA signature and highlights the importance of its further evaluation [15]. In addition, the remaining eight differentially expressed miRNAs are involved in the regulation of key pathological processes, including apoptosis, inflammatory responses, axonal elongation, and neuroprotection, underscoring the need for their comprehensive functional characterization in the context of SCA7 pathogenesis [20,21].

### 4.4. Extraction of miRNAs from Plasma Samples and Retrotranscription

Total RNA was isolated from 200 µL of plasma using the miRNeasy serum/plasma kit (Qiagen, Hilden, Germany) following the manufacturer’s instructions. The RNA obtained was eluted in 30 µL of nuclease-free water. The concentration of total RNA was quantified by spectrophotometry using the Nano Drop 2000 equipment (Thermo Fisher Scientific, Walthman, MA, USA). RNA samples were stored at −80 °C.

The reverse trascription (RT) was performed using a concentration of 60 ng of total RNA, with the RT-reverse transcriptase kit (Taqman ^TM^ MicroRNA Reverse Transcription Kit; Thermo Fisher Scientific, Walthman, MA, USA) and specific primers (taqman miRNA probes, Thermo Fisher Scientific, Walthman, MA, USA), we used adjustments based on the Kroh protocol [69] in the Gene Amp 9700 PCR system thermocycler (Thermo Fisher Scientific, Walthman, MA, USA) with the following conditions: 16 °C during 30 min, 42 °C during 30 min and 85 °C during 5 min.

From the cDNA obtained, a preamplification reaction was performed using the PreAmp Megaplex™ primers and the TaqMan^®^PreAmp Kit PreAmpMasterMix mix (Thermo Fisher Scientific, Walthman, MA, USA). Following the manufacturer’s instructions, the sample was subsequently treated as follows: 40 cycles at 16 °C during 2 min, 42 °C during 1 min, and 50 °C during 1 s followed by 80 °C during 5 min for the inactivation of the enzyme. The reaction was carried out in the Gene Amp 9700 PCR System Thermal Cycler (Thermo Fisher Scientific, Walthman, MA, USA). Subsequently, the cDNA was stored at −20 °C for further use.

### 4.5. Relative Quantification of miRNAs

The quantitative PCR (qPCR) was performed taking the preamplification product as template and using the assays specific for miRNAs: hsa-miR-215-5p, hsa-miR-342-5p, hsa-miR-365a-3p, hsa-miR-375-3p, hsa-miR-17a-5p, hsa-miR-18a-5p, hsa-miR-19a-3p, hsa-miR-20a-5p, hsa-miR-25-3p, hsa-miR-92a-3p, miR-93-5p, hsa-miR-106a-5p (Thermo Fisher Scientific, Walthman, MA, USA) each reaction was performed in duplicate, and the hsa-miR-483 was used as an endogenous control [ID002338] [15]. For plasma samples, hsa-miR-483 was used as the endogenous control due to its demonstrated stability in plasma and its ability to preserve amplification efficiency in samples with low amounts of genetic material [15], as other commonly used endogenous controls often exhibit significant interindividual variability. In contrast, in cell cultures, transcription levels can vary markedly due to factors such as cell cycle progression, confluence, or metabolic stress [70]. Therefore, different reference genes (RNU44 [ID001094], RNU48 [ID0493161], and U6 [ID001973]) were used to normalize these biological fluctuations in the glial cell model, as previously reported.

The cycling of qPCR was performed in triplicate on the StepOne-Real Time PCR system (Thermo Fisher Scientific, Walthman, MA, USA). The amplification conditions were: 50 °C for 2 min, 95 °C for 10 min (denaturation), and 40 cycles of 95 °C for 15 s, 60 °C for 60 s.

The relative expression levels of miRNAs were calculated using the comparative Delta Delta Ct (∆∆Ct) method [71]. The Ct value was determined at baseline and at a threshold of 0.2 fluorescence units on the plates. Relative miRNA expression was calculated using the comparative ΔΔCt (2^−ΔΔCt^) method. Briefly, cycle threshold (Ct) values of each miRNA were first normalized to the geometric mean of the selected endogenous gene controls to obtain ΔCt (ΔCt = Ct_miRNA − Ct_reference). Subsequently, ΔΔCt was calculated by subtracting the ΔCt of the age- and sex-matched healthy control (calibrator) from the ΔCt of the corresponding sample (ΔΔCt = ΔCt_sample − ΔCt_calibrator). Log2 fold change (log2FC) was then obtained as −ΔΔCt, providing a symmetric measure of relative up- or down-regulation with respect to the paired control. miRNA with a *p*-value < 0.05 and a log2 fold change (log2FC) factor > 1 was considered as differentially expressed.

### 4.6. Cell Culture of the Stable Model of Müller Glia in Retina Based on MIO-M1 Cells for SCA7

Previously, our group developed and validated a cellular model for SCA7-inducible retinal glia based on MIO-M1 cells, which expresses the *ATXN7* gene with 10 CAG repeats (control) or the *ATXN7* gene as a model of SCA7 with 64 CAG repeats, under the control of a promoter that is doxycycline (Dox) inducible [46]. Glial cells in our cellular model were cultured in six-well plates at 37 °C and 5% CO_2_ in Dulbecco’s Modified Eagle Medium (DMEM) (Invitrogen, Carlsbad, CA, USA), supplemented with 10% fetal bovine serum (FBS), 100 U/mL penicillin, 100 µg/mL streptomycin, 350 µg/mL geneticin (G418), and 0.35 µg/mL puromycin. Once the cell confluency reached 70%, the induction of the *ATXN7* transgene was carried out by incubating the cells with 1 µg/mL of Dox, and then the cells were harvested at 24 h, 48 h, 72 h and 96 h post-induction. The harvested cells were treated with Qiazol for subsequent extraction of miRNAs, using the miRNeasy kit (Qiagen, Hilden, Germany) according to the manufacturer’s instructions. The genetic material was stored at −80 °C for later use. The presence of miRNAs in every culture was evaluated in triplicate by qRT-PCR.

### 4.7. Analysis of miRNAs Interaction with Target Genes

In order to predict the potential target genes of miRNAs, we used the miRNet V2.0 platform. This platform allowed us to analyze a network aimed to identify target miRNA-mRNA interactions, based on prediction and experimental findings, which is included in the TARBase V9.0 base [72,73]. Also, to integrate the pathway enrichment results, we used the Kyoto Encyclopedia of Genes and Genomes (KEGG) database and the genes involved in the signaling pathways were selected to generate an interaction network through miRNet V2.0 and Cytoscape V3.10.1 software.

### 4.8. Statistical Analysis

Descriptive statistics were applied to the clinical and demographic characteristics of the study groups, which are represented as means and standard deviations (SDs). The relative expression levels of miRNAs were calculated using the comparative delta–delta Ct method (∆∆Ct) [71].

The normality of the data was assessed using the Shapiro–Wilk test, the comparisons of the miRNA expression in plasma between groups was determined using the non-parametric Mann–Whitney U test, while the comparison expression of the miRNAs from the SCA7 cell model was performed using a repeated-measures ANOVA with post hoc Tukey; the GraphPad Prism version 10 statistical package was used, and a significant difference was considered when *p* < 0.05.

Differential expression between EO and AO groups was assessed using log2FC values. Data distribution for each miRNA was evaluated using normality and lognormality tests, and group comparisons were performed using Welch’s *t*-test or the Mann–Whitney U test as appropriate. Receiver operating characteristic (ROC) curve analysis was conducted to evaluate the discriminative performance of individual miRNAs between EO and AO groups by assessing sensitivity and specificity; Area Under the Curve (AUC), standard error, 95% confidence intervals, and *p*-values were calculated using a non-parametric DeLong approach implemented in R. Associations between miRNA expression levels and clinical or molecular variables, including CAG repeat length, age at onset, and S factor, were assessed using Spearman’s rank correlation coefficient with false discovery rate correction. Statistical significance was set at *p* < 0.05. Graphical visualization and complementary statistical analyses were performed using GraphPad Prism version 10.6.1.

## Figures and Tables

**Table 1:** General characteristics of study individuals.

Characteristics	Healthy Individuals (*n* = 16)	SCA7 Patients (*n* = 16) [AO (*n* = 8); EO (*n* = 8)]
**Sex *n* (%)**		
Male	12 (75%)	12 (75%)
Female	4 (25%)	4 (25%)
**Age (year)**		
Mean ± SD	38.93 ± 13.89	35.75 ± 13.86
Median (range)	38 (18–66)	35.5 (11–66 years)
**Number of CAG repeats**		
Mean ± SD	10.25 ± 0.87	49.75 ± 9.22
Median	10	46.5
CAG repeats range	(10–12)	(39–72)
Disease duration (year)		9.81 ± 14

## Data Availability

The data presented in this study are available on request from the corresponding authors.

## Supplementary Materials

The following supporting information can be downloaded at: https://www.mdpi.com/article/10.3390/ijms27020683/s1.
