# Supplementary material for: Integrated Plasma and Glial Cell Evidence Indicates a Functional Role for hsa-miR-342-5p in Spinocerebellar Ataxia Type 7 and Its Potential Use as a Biomarker
Source: Int J Mol Sci. 2026 Jan 9;27(2):683. doi: 10.3390/ijms27020683 (PMC12840712; doi:10.3390/ijms27020683)
Supplement: Supplementary file 1 [file ijms-27-00683-s001.zip › ijms-4046844-supplementary.pdf]

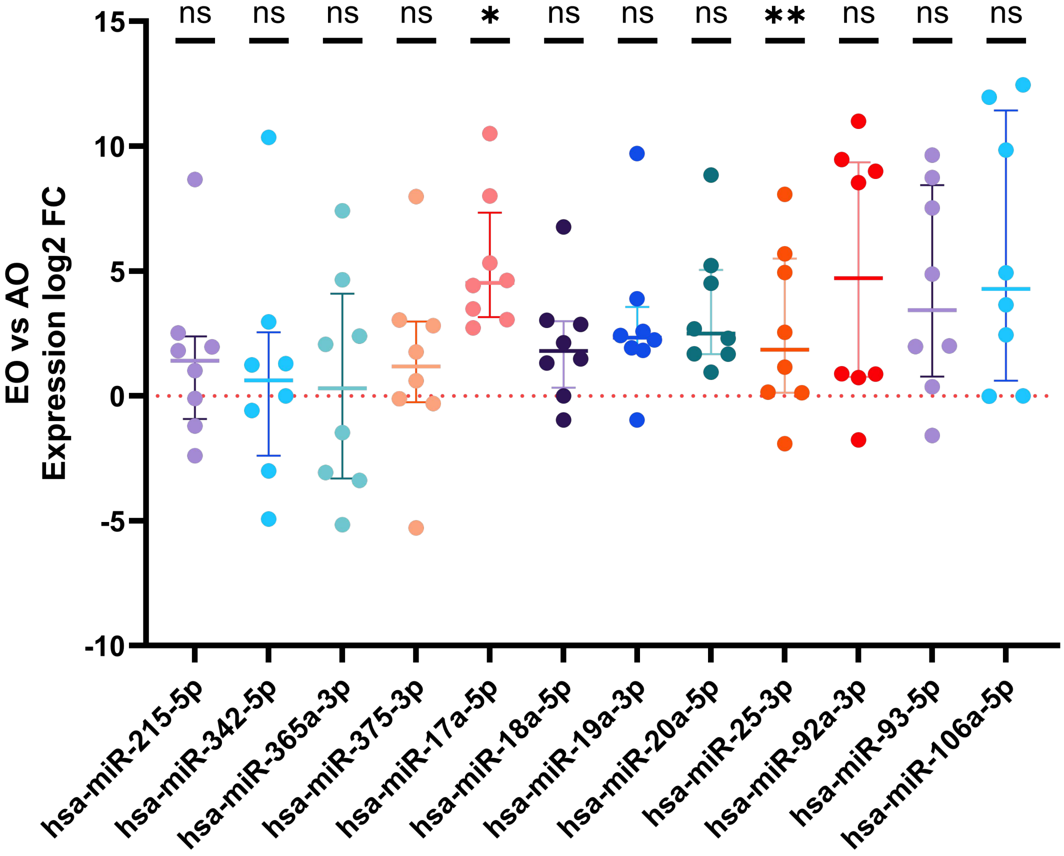

Supplementary Figure S1. Relative miRNA expression levels in the plasma (log_2 fold change) between early-onset (EO) and adult-onset (AO) SCA7 patients. The data set, encompassing twelve miRNAs, is presented as medians and interquartile ranges. Results are shown as log2 fold change (Log2FC), where positive and negative values indicate increase or decrease relative expression to the reference group. Log2FC greater than 1 is considered as change. Group comparisons were performed using Welch's t-test or the Mann–Whitney U test, as appropriate. A p-value < 0.05 was considered significant, asterisks denote significant differences between EO and AO (* $p < 0.0332$; ** $p < 0.007$). The study included 8 subjects in each group. ns = no significant.

Supplementary Table S1. Correlation analysis between miRNA expression levels and clinical and genetic variables in SCA7 patients

	CAG	Age of Onset	S-Factor
**SCA7**	***r (p)***	***r (p)***	***r (p)***
hsa-miR-215-5p	-0.22 (0.414)	0.24 (0.376)	-0.17 (0.528)
hsa-miR-342-5p	0.15 (0.573)	-0.11 (0.691)	-0.13 (0.640)
hsa-miR-365a-3p	0.08 (0.770)	-0.06 (0.828)	-0.15 (0.587)
hsa-miR-375-3p	-0.01 (0.957)	0.05 (0.854)	-0.04 (0.871)
hsa-miR-17a-5p	0.26 (0.326)	-0.31 (0.251)	0.27 (0.316)
hsa-miR-18a-5p	-0.09 (0.749)	0.04 (0.892)	0.26 (0.327)
hsa-miR-19a-3p	0.00 (0.998)	-0.07 (0.797)	0.12 (0.668)
hsa-miR-20a-5p	0.05 (0.841)	-0.08 (0.757)	-0.16 (0.549)
hsa-miR-25-3p	0.61 (0.013)	-0.63 (0.009)	0.33 (0.208)
hsa-miR-92a-3p	0.22 (0.420)	-0.26 (0.323)	0.29 (0.274)
hsa-miR-93-5p	-0.32 (0.229)	0.34 (0.199)	-0.06 (0.837)
hsa-miR-106a-5p	-0.16 (0.552)	0.09 (0.745)	0.16 (0.549)
**AO**			
hsa-miR-215-5p	-0.61 (0.105)	0.57 (0.143)	-0.52 (0.183)
hsa-miR-342-5p	-0.37 (0.362)	0.22 (0.606)	-0.40 (0.320)
hsa-miR-365a-3p	0.10 (0.820)	0.00 (1.000)	-0.19 (0.651)
hsa-miR-375-3p	-0.11 (0.798)	0.16 (0.711)	-0.12 (0.779)
hsa-miR-17a-5p	-0.36 (0.379)	0.29 (0.487)	0.21 (0.610)
hsa-miR-18a-5p	-0.30 (0.468)	0.27 (0.526)	0.29 (0.493)
hsa-miR-19a-3p	0.31 (0.450)	-0.29 (0.487)	0.21 (0.610)
hsa-miR-20a-5p	0.05 (0.910)	-0.05 (0.910)	-0.31 (0.456)
hsa-miR-25-3p	0.27 (0.526)	-0.25 (0.545)	0.33 (0.420)
hsa-miR-92a-3p	0.29 (0.487)	-0.31 (0.450)	0.43 (0.289)
hsa-miR-93-5p	-0.13 (0.754)	0.16 (0.711)	0.21 (0.610)
hsa-miR-106a-5p	-0.37 (0.362)	0.29 (0.487)	-0.07 (0.867)
**EO**			
hsa-miR-215-5p	0.25 (0.548)	-0.08 (0.844)	-0.14 (0.736)
hsa-miR-342-5p	0.20 (0.627)	0.09 (0.831)	0.02 (0.955)
hsa-miR-365a-3p	-0.61 (0.108)	0.57 (0.136)	0.12 (0.779)
hsa-miR-375-3p	-0.04 (0.933)	0.17 (0.691)	0.14 (0.736)
hsa-miR-17a-5p	-0.18 (0.670)	0.04 (0.933)	0.14 (0.736)
hsa-miR-18a-5p	0.32 (0.435)	-0.47 (0.243)	0.02 (0.955)
hsa-miR-19a-3p	-0.56 (0.146)	0.30 (0.471)	0.00 (1.000)
hsa-miR-20a-5p	-0.36 (0.382)	0.24 (0.568)	-0.07 (0.867)
hsa-miR-25-3p	-0.06 (0.888)	-0.07 (0.866)	0.14 (0.736)
hsa-miR-92a-3p	-0.79 (0.020)	0.59 (0.126)	0.24 (0.570)
hsa-miR-93-5p	-0.81 (0.014)	0.85 (0.007)	-0.14 (0.736)
hsa-miR-106a-5p	-0.73 (0.040)	0.49 (0.217)	0.05 (0.911)

Results are presented as Spearman's correlation coefficient *r* followed by the corresponding p-value in parentheses *(p)*.
